# A Digital Microintervention Supporting Evidence-Based Parenting Skills: Development Study Using the Agile Scrum Methodology

**DOI:** 10.2196/54892

**Published:** 2024-06-28

**Authors:** Nathan Hodson, Peter Woods, Michael Sobolev, Domenico Giacco

**Affiliations:** 1 Price School of Public Policy University of Southern California Los Angeles, CA United States; 2 Mental Health and Wellbeing Warwick Medical School University of Warwick Warwick United Kingdom; 3 Cedars-Sinai Medical Center Los Angeles, CA United States; 4 Schaeffer Center for Health Policy and Economics University of Southern California Los Angeles, CA United States

**Keywords:** parenting, child behavior, mental health, app development, digital

## Abstract

**Background:**

Conduct disorder increases risks of educational dropout, future mental illness, and incarceration if untreated. First-line treatment of conduct disorder involves evidence-based parenting skills programs. Time-outs, a frequent tool in these programs, can be effective at improving behavior, and recent apps have been developed to aid this process. However, these apps promote the use of time-outs in inconsistent or developmentally inappropriate ways, potentially worsening behavior problems. Digital microinterventions like these apps could guide parents through high-quality time-outs in the moment, but current time-out apps lack features promoting adherence to the evidence-based best practice. Agile scrum is a respected approach in the software development industry.

**Objective:**

We aimed to explore the feasibility of using the agile scrum approach to build a digital microintervention to help parents deliver an evidence-based time-out.

**Methods:**

The agile scrum methodology was used. Four sprints were conducted. Figma software was used for app design and wireframing. Insights from 42 expert stakeholders were used during 3 sprint reviews. We consulted experts who were identified from councils around the Midlands region of the United Kingdom and charities through personal contacts and a snowballing approach.

**Results:**

Over 4 development sprints from August 2022 to March 2023, the app was iteratively designed and refined based on consultation with a diverse group of 42 experts who shared their knowledge about the content of common parenting programs and the challenges parents commonly face. Modifications made throughout the process resulted in significant app enhancements, including tailored timer algorithms and enhanced readability, as well as an onboarding zone, mindfulness module, and pictorial information to increase inclusivity. By the end of the fourth sprint, the app was deemed ready for home use by stakeholders, demonstrating the effectiveness of our agile scrum development approach.

**Conclusions:**

We developed an app to support parents to use the evidence-based time-out technique. We recommend the agile scrum approach to create mobile health apps. Our experience highlights the valuable role that frontline health and social care professionals, particularly those working with vulnerable families, can play as experts in scrum reviews. There is a need for research to both evaluate the impact of digital microinterventions on child behavioral change and also create digital microinterventions that cater to non–English speakers and individuals who participate in parenting programs in settings outside the United Kingdom.

## Introduction

### Background

Conduct disorder is the most common mental health disorder of childhood, and around 5% of children meet diagnostic criteria [1,2]. Children with conduct disorder persistently violate the rights and boundaries of adults and other children, typically by breaking serious rules, being aggressive in ways that cause harm, and stealing or damaging property [3]. Untreated, children with conduct disorder face increased risk of educational drop-out, future mental illness, and incarceration [4,5]. But with treatment their long-term outcomes can be improved [6,7].

The best treatment for conduct disorder is to offer parents a parenting skills program [8]. Systematic review evidence shows that programs like Triple P, Incredible Years, and the Chicago Parenting Program can equip parents to change their parenting style, change their child’s behavior, and improve their long-term prospects [6,9]. These programs, usually aimed at the parents of children aged 2-10 years, use a range of teaching modalities to equip parents to improve their relationship with their child and then to set consistent and appropriate limits [10]. This change in parenting style allows children to develop healthy regulation of their own emotions, although converting classroom learning into parenting style change at home can be difficult, in keeping with the psychological theory of the hot-cold empathy gap [11].

Mobile technology may provide a means of better facilitating this change. The development of mobile apps for health conditions like conduct disorder remains a novel field. But approaches from the software development industry, such as agile scrum, may provide useful structure to the co-design and feedback process in order to produce high-quality user experiences [12].

### Time-Outs

Time-outs (or an equivalent such as quiet time or calming time) are a common element of these parenting skills programs [13]. In time-outs, the parent asks a child to sit quietly for 3-10 minutes in response to a breach of family rules, such as aggression or persistent refusal to follow instructions. An extensive evidence base has described the characteristics of an effective time-out, drawing on a large number of intervention studies conducted over the last 40 years [14]. These features include a safe and nonstimulating environment, an appropriate reason for initiating the time-out, a short and developmentally appropriate duration, parental control of the end of the time-out, and consistent rules that are codified by parenting skills programs as simple protocols for parents to follow [13].

Recent evidence shows that 85% of parents who use time-outs fail to deliver evidence-based time-outs, often setting the duration for too long, using it for an inappropriate reason, or ending time-outs with lecturing and criticism [14]. For these families, failure could mean child behavior becomes worse rather than better. For children affected by conduct disorder, half-hearted, overly harsh, or inconsistent time-outs could mean they fail to develop the self-regulatory skills required to thrive in adolescence and adulthood [5,13].

Parenting skills programs increasingly draw on digital technology to maximize their reach and impact [15]. The domain of time-outs is no exception, but a review of all 6 time-out apps available on the App Store, Play Store, and Alexa found none had features promoting evidence-based time-out skills [16]. Although they all supported consistency, many created a stimulating environment, ended time-outs prematurely, or offered inappropriate durations [16]. In many domains there was an absence of advice for parents (regarding appropriate reasons and durations, for example) and in others the apps made it harder to follow guidelines (eg, by including cartoons and music during time-outs) [16]. Parenting skills programs have been, therefore, unable to recommend any time-out apps to parents seeking guidance [16,17]. We involved stakeholders in an agile scrum approach to co-design an app that would overcome these limitations.

Time-out apps are digital microinterventions. Baumel et al [18] defined digital microinterventions as “highly focused interventions delivered in the context of a person’s daily life with little burden on the individual.” When parents use a time-out app during a child behavior crisis, the guidance on the app directly informs parents about how to deliver a time-out. Digital microinterventions do not demand burdensome book learning or protocol memorizing, but give parents advice in the moment. Time-outs are a specific short task with a strong evidence base and as such are appropriate for the development of a mobile app digital microintervention to support parents [16].

### Agile Scrum

Agile is the industry standard iterative product management approach to app design [19]. The Agile Manifesto outlines the principles of agile development, including a focus on working features rather than creating a perfect final product and an emphasis on listening to clients rather than being fixated on protocols [20]. Agile is characterized by short cycles of development called sprints rather than taking a “waterfall” approach of working toward a final product from the start of the project. Insights from the agile philosophy have also informed the development of health behavior change interventions [19].

Scrum is an agile framework specifically designed for software development. This framework for applying the agile approach helps to define the steps required to apply the agile values in software development. First developed by Sutherland and Schwaber [12], scrum defines key roles and key people in the process, which includes three stages to conduct a sprint using scrum: (1) a product owner orders the work for a complex problem into a product backlog; (2) the scrum team turns a selection of the work into an increment of value during a sprint; (3) the scrum team and its stakeholders inspect the results and adjust for the next sprint.

As health care increasingly involves drawing on software and mobile apps, it is also valuable to find out whether agile scrum is a practicable approach to developing other future health apps. This is the first published report of how agile scrum has been used to make a parenting app. Our aim was to build a digital microintervention app to help parents deliver evidence-based time-outs using the agile methodology.

## Methods

### Approach and Development

We used the agile scrum framework to structure an iterative, stakeholder-led development process in keeping with the Agile Manifesto [20]. We used Figma (Figma Inc) for app design and wireframing. For iOS, coding was executed using SwiftUI in Xcode (Apple Inc). For Android, development was completed using Kotlin and XML in Android Studio (Google LLC).

### Expert Stakeholders

We consulted 42 parenting experts in our sprint reviews. Experts were contacted via councils around the Midlands region of the United Kingdom and national charities through personal contacts and a snowballing approach. Experts had experience of diverse professions involved in supporting children with conduct disorders, including psychiatry, family nursing, and parenting skills program provision (Table 1). All experts were involved in providing frontline care to families impacted by disrupted behavior problems. Experts were only invited if they were employed full time in providing parenting skills support to families. We did not require all experts to attend every sprint review due to the other demands on their time.

**Table 1:** Experts (N=42).

Organization		Experts, n
Charities		3
**Local government parenting skills teams**		
	Local authority 1	3
	Local authority 2	8
	Local authority 3	4
	Local authority 4	1
Mental health staff		4
Family nursing		8
Other parenting teams		11

We chose to consult with experts rather than parents for several reasons. First, these experts are gatekeepers for recommending the app, so people in their position would be the primary users. Second, experts work closely with a large number of parents at a variety of stages of their journey, focusing specifically on the topic at hand and providing troubleshooting support, so they bring a wider understanding of the diverse range of programs and possible user stories than we would be able to assemble from a panel dominated by parents for whom personal experience would be more salient. Third, the majority of included experts were also parents and could combine their own experiences with their professional expertise. Finally, this choice ensured that suggestions made by experts provided insights into the parenting skills evidence base and best practices, which would not have been the case if nonexperts were relied upon.

### Sprint Review

Experts inspected the results of the development sprint during the sprint review. The sprint review began with presentation of the latest features of the app. Experts were permitted to download the app to explore the features and use it with their own families if they wished. Experts who had young children living with them were able to use the app with their own families. We did not record how frequently they did so, and no data were collected on how they used the app. Some experts shared the app with friends or with parents with whom they were working one-to-one. Experts were prompted to work through the app, reviewing the way the app architecture fitted with the ethos of their parenting programs and the challenges faced by parenting skills coaches. The key results of the reviews were new problems identified in the way the app would be used by parents that they would want to be improved. Sprint reviews were conducted in different settings (face to face, over audio, or over video) as suited the experts. Reviews were only minuted, they were not audio- or video-recorded. Some additional feedback was received via email.

The discussions at the sprint review were used to generate a new backlog. Specifically, the backlog was generated from the list of user stories, which was honed and updated after each sprint review. The changes made included either changes to existing user stories or additions of new user stories. This backlog was created through a form of modified narrative analysis whereby new user stories were developed, contested, and cocreated with experts during reviews [21,22]. The user stories related to the end users, that is, parents attending parenting groups designed and provided by the expert stakeholders, not the expert stakeholders themselves. The expert stakeholders were ideally placed to share insights of relevance to the end users because designing and providing parenting programs affords them extensive appreciation of the lives of the end users, particularly as they relate to challenges managing behavior.

Analysis of sprint reviews focused on user stories regarding the gains and pains for users, rather than specifying the features required to achieve those goals [23]. The other theoretical foundation for development of user stories was the Easy, Attractive, Social, and Timely (EAST) framework from the behavioral insights team, which simplifies the process of applying findings from behavioral economics to real-world problems by promoting 4 principles: easiness, attractiveness, social factors, and timing [24]. This framework was used to help designers build behaviorally informed solutions based on the user stories generated. We also drew on the expert knowledge of stakeholders regarding changes to timing and phrases in the app.

There were two possible termination conditions: (1) experts stated that they would offer the app to their clients, or (2) experts reported the app could not be improved.

### Ethical Considerations

The UK Research and Innovation Medical Research Council tool found that this study is not research as defined by the UK Policy Framework for Health and Social Care Research and the Biomedical Sciences. The Research Ethics Committee at University of Warwick confirmed that formal ethical approval was not required [25]. Experts were offered vouchers for contributing but none accepted.

## Results

Four sprints took place between August 2022 and March 2023. The description below summarizes the results from each sprint scrum.

### Initial Design: Sprint 1

Sprint 1 began with the following example user story: “As an Incredible Years user I want a reminder of how to get time out right so me and my partner keep it consistent every time.” This example user story arose from the challenge faced by one of the researchers who is an Incredible Years–trained coach but noticed no apps were available to help parents. This user story led to the generation of a backlog. The goal was to create an Android app that would include a timer parents could use during time-outs. Before launching the timer, there would be gates for parents to go through where they confirm that they are using time-outs for an evidence-based reason. Parents would have to enter the child’s age to set the timer, and this would be used to screen for misuse of time-outs with children who are too old or too young. To start the time-out, parents would be prompted to ensure their child was in a time-out space and was quiet. They would have the option to access information about what to do if their child was escaping the time-out space. At the conclusion of the time-out, parents would have the option of viewing advice about how to use effective praise with their child.

### Usability Testing: Sprint Review 1

Sprint 1 concluded with a sprint review where 10 experts were consulted, and new user stories were generated. New user stories included “I was trained at another parenting program and I want a time out guide that fits with what I learned from my course”; “I don’t know where to start with time out at home”; “My child is not developmentally ready for a time out so I want to make the time shorter”; and “When I set the app I am in a moment of stress so I need it to be easy to read the information.”

### Sprint 2

These new user stories drove the development of a backlog for sprint 2. The backlog included 2 new timer algorithms with different instructions and decision rules tailored for the guidance of other popular parenting skills programs. Some of the language in the app was changed for compatibility with a wider range of parenting skills programs. It was possible for parents to set timers with or without using the child’s age. The user experience was redesigned for ease of reading and better labeling of buttons. The color scheme was also completely changed. Checklists were designed to help parents prepare for a time-out by explaining the process to their child and identifying an appropriate time-out place.

Sprint 2 concluded with sprint reviews where we obtained guidance from 16 experts, and the following new user stories were developed: “As a parenting skills coach I want to be able to choose which settings parents see as this will reduce confusion;” “As a parent, time out is stressful. I want something to help me and my child calm down so it is more likely to end well”; “Another parent told me time out is bad for children so I’m not interested in this app”; and “Sometimes I see amazing moments of connection in parents but they don’t always notice their successes.”

### Sprint 3

The backlog for sprint 3 included the creation of an onboarding zone, which parents would only encounter during their first login. This was intended to give parenting skills coaches the opportunity to help parents choose the settings that would suit them best. We added a visual prompt for mindful breathing during the time-out to help parents calm down and to allow them to engage in calming, mindful breathing together with their child if they wished. This feature allowed parents to use an emotion-coaching activity when it could be tolerated, instead of only allowing a time-out with negative reinforcement. Some text was rephrased to help parents to notice their successes after the conclusion of the time-out, including notifications to remind parents of their successes 15 minutes after the timer ended. The term “time-out” was also taken out of the app title and made less prominent in response to concerns among experts that it was too narrow.

After sprint 3 was completed, a sprint review was conducted drawing on insights from 24 of the experts. New user stories were generated: “When everyone is upset it is sometimes hard to click through questions and I would like to skip questions”; “My child likes using the mindfulness before bed to calm down, but I feel bad setting the time-out timer”; “Many of our parents speak Polish or Ukrainian better than English and would feel included by pictorial information”; and “I decided to go through the app without my children present so I could deliver a time-out without having my phone out.”

### Final Design: Sprint 4

The backlog for sprint 4 included a separate mindfulness module to incorporate more mindfulness activities and encouraged parents to use the app’s mindfulness activities for practice. A “mini-timer” button was added to the home page to allow parents to use their discretion to skip the checking questions in a moment of crisis. We were alert to the risk this would mean parents overlooked some other evidence-based principles of time-outs, but experts advised that it would be important to give this option to ensure parents and other children were safe during rapidly escalating situations. Specifically, this would ensure that parents were not distracted by a complicated user interface in the event that a child was being immediately violent toward another family member, requiring prompt intervention. Several stages were removed from the app to make it quicker for parents to navigate the app both by removing unnecessary confirmations and redesigning to allow more options on each page. Icons were added to help people who could not read English.

A sprint review was conducted, attended by 15 experts, where parenting skills coaches from different locations reported that they would offer the app to parents in a research setting. As such the termination conditions were met, and no further sprints were required. Textbox 1 summarizes the list of features the app included at the end of the final sprint.

**Final design features.**

**Timers**

- Visual countdown timer
- Mindfulness exercise during timer
- Parents supported to use praise after timer
- Age-appropriate duration set
- Countdown when child is sitting and quiet
- Check appropriate reason for timer
- Guide to different problems (breaking the rules, refusing instructions)
- Suggests ignoring or distraction if required
- Advice for handling running away or shouting

**Navigation**

- Mini-timer without checking questions for crisis situation
- Choice of timers at launch
- Home button added throughout app
- Easy to read aesthetic with simple sentences in large text

**Information**

- 4 subsections with guidance about giving effective praise
- Explanation about why praise is important
- Information about when to consider using a time-out

**Checklists**

- Guide to explaining time-outs to children
- Guide to preparing a safe time-out space

## Discussion

### Summary of Findings

The aim of this study was to create an interactive app assisting parents in implementing time-outs at home using an agile scrum approach. Over 4 development sprints from August 2022 to March 2023, the app was iteratively designed and refined based on guidance from a diverse group of 42 experts. Modifications made throughout the process resulted in significant app enhancements, including tailored timer algorithms, enhanced readability, an onboarding zone, a mindfulness module, and pictorial information to increase inclusivity. By the end of the fourth sprint, the app was deemed ready for home use by experts, illustrating that the industry standard agile scrum development approach was feasible.

### Comparison With the Literature

Other mental health apps have benefited from using agile, including agile scrum, but none related specifically to parenting skills training [26-30]. Bucci et al [31] called for researchers to take a coproduction approach to the development of mental health technology. But there have been very few reported coproduction initiatives in child and adolescent mental health technology [32]. Agile scrum methodology creates a structure that centers stakeholders throughout the app development process; when these stakeholders are frontline experts, as in this case, or even patients, this process becomes one of co-design.

While other approaches to co-design exist, such as generative research design and transformation design, agile scrum brings together both app development and co-design smoothly and efficiently [33,34]. Although a review of all co-design approaches is beyond the scope of this paper, it is illustrative to consider how agile scrum differs from existing approaches. Generative research design involves creating selection systems from which users themselves create the final design, whereas our approach and transformation design bring together diverse stakeholders and users in order to address social problems [33,34]. Unlike generative research design, the product owner in agile scrum must bring contextual knowledge and integrate this with the stakeholder feedback in order to generate new user stories (in this case, through the modified narrative analysis process). Unlike transformation design, agile scrum formalizes an iterative loop connecting the work of the developers and the experience of stakeholders.

The resulting time-out app is the first app to guide parents toward only delivering time-outs when there is a good reason, the first app to support parents to control the end of time-outs, and the first app to encourage parents to use praise with time-outs [16]. This agile scrum process has therefore produced a more useful digital microintervention than existed before. Baumel et al [18] argued that digital microinterventions are best conceived as building blocks of large therapeutic interventions. By drawing on the experience of parenting skills coaches, our agile scrum approach ensured the app was well-suited to contribute to a comprehensive parenting skills program that would involve a range of other skills and social support [10].

Other web-based parenting skills programs primarily change child behavior by using web-based modules or video chat groups to teach parents new styles and techniques [10,35]. This common approach relies on parents recalling and implementing learning. Parent-child interaction therapy, a well-supported approach to parenting style change, frequently uses a “bug-in-ear” approach allowing coaches to give parents live guidance as they interact with their children during therapy sessions [36,37]. The app developed during this program mirrors this bug-in-ear approach by communicating evidence-based techniques while parents engage with children.

### Strengths and Limitations

This is one of the first studies to report the use of agile scrum in health care app development [12]. This approach facilitated a feedback loop in the design process that allowed the app to be continuously refined across 4 development sprints, with 3 main benefits. First, this approach meant it was easy to pivot to create an app that could serve parents in different parenting programs. Second, this approach meant bugs could be identified early. Third, the process remained focused on stakeholders. The diverse group of 42 expert stakeholders ensured development was oriented toward solving real-world problems. By returning to expert stakeholders over subsequent sprints, they became invested in the project and their depth of engagement and quality of feedback increased. The principle behind the app—a digital microintervention—is another strength. Digital microinterventions are intended to capitalize on the ubiquity of smart phones and have the potential to impact behavior in everyday contexts but have not been widely developed [18]. Finally, continuous efficacy testing was not required given the extensive body of research showing that disruptive behavior problems are improved by parenting skills coaching, including high-quality time-outs [6,13].

Despite these strengths, the small amount of parent testing was a limitation to the study. Although anecdotally some parenting skills coaches used the app with their own children, their primary interest in the app related to their coaching role. This means we have limited information on the practical use of the app at home. This agile scrum approach may benefit in future from including nonexpert populations among the stakeholders to further improve user experience, but we suggest that drawing on insights from frontline experts rather than users is preferable in a mental health setting where end users are vulnerable. Additionally, the app was not formally tested for effectiveness or risks. Even though it provides evidence-based guidance on improving time-out quality, which is known to improve disruptive child behavior, it is possible that it interacts with family life in other ways to reduce these gains, possibly by increasing parent phone use [13,16]. In addition, parents’ busy and stressful lives can undermine their engagement with any type of mobile health app [38,39]. Directly studying child behavior and parents’ engagement with the app would be informative and will be addressed in a user experience study [40].

The study was limited to the Midlands region of the United Kingdom and the app was only available in English. Although the Midlands is a diverse area, it would be valuable to evaluate the acceptability of the app elsewhere in the world and in other languages.

Finally, the agile scrum approach was highly resource intensive, requiring large amounts of developer time, repeated stakeholder contacts, careful sequencing of these events, and multiple meetings communicating user stories and backlog. A waterfall approach would have been quicker and cheaper but would not have achieved the same match between the resulting app and the expert stakeholders’ expectations, given our initial expectations were somewhat different from the end product.

### Implications for Policy, Practice, and Research

We have generated a digital microintervention mobile app to support parents to deliver evidence-based time-outs. We recommend that researchers aiming to create apps that enhance parenting skills or promote children’s mental health adopt the agile scrum approach. We have described the strengths of this approach and highlighted pitfalls that those adopting this approach in the future should attempt to mitigate. Our findings highlight the valuable role that frontline health and social care professionals, particularly those working with vulnerable families, can play as expert stakeholders in scrum. Further research should evaluate the impact of digital microinterventions on child behavioral change and also create digital microinterventions that cater to non-English speakers and individuals who participate in parenting programs in settings outside the United Kingdom. Significantly, this study underscores the feasibility of agile scrum in creating parenting skills apps that can seamlessly integrate into parenting skills coaching, and we encourage researchers to explore how similar apps can streamline parenting programs and draw on insights from behavioral science [41]. Continuing to use technology to make it easier for parents to use evidence-based techniques will ultimately enhance the prospects of children with disruptive behavior disorders and improve their family lives.

### Conclusion

We developed an app to support parents to use the evidence-based time-out technique. We recommend the agile scrum approach to create mobile health apps. Our experience highlights the valuable role that frontline health and social care professionals, particularly those working with vulnerable families, can play as experts in scrum reviews.

## Abbreviations

EAST: Easy, Attractive, Social, and Timely
